# In-feed nutritional additive probiotic *Saccharomyces boulardii* RC009 can substitute for prophylactic antibiotics and improve the production and health of weaning pigs

**DOI:** 10.14202/vetworld.2023.1035-1042

**Published:** 2023-05-15

**Authors:** Julián Parada, Alejandra Magnoli, Maite Corti Isgro, Valeria Poloni, Analía Fochesato, María Pía Martínez, Alicia Carranza, Lilia Cavaglieri

**Affiliations:** 1 Department of Animal Pathology, Faculty of Agronomy and Veterinary, National University of Rio Cuarto, Río Cuarto, Córdoba, Argentina; 2 National Scientific and Technical Research Council (CONICET), Córdoba, Argentina; 3 Department of Animal Production, Faculty of Agronomy and Veterinary, National University of Rio Cuarto, Río Cuarto, Córdoba, Argentina; 4 Department of Microbiology and Immunology, Faculty of Exact, Physical, Chemical and Natural Sciences, National University of Río Cuarto, Río Cuarto, Córdoba, Argentina

**Keywords:** nutritional feed additive, post-weaning pig, prophylactic antibiotics, *Saccharomyces cerevisiae* var. *boulardii* RC009

## Abstract

**Background and Aims:**

Non-therapeutic antibiotic use is associated with the current decrease in antibiotic therapeutic efficiency and the emergence of a wide range of resistant strains, which constitutes a public health risk. This study aimed to evaluate the use of *Saccharomyces cerevisiae* var. *boulardii* RC009 as a nutritional feed additive to substitute the prophylactic use of antibiotics and improve the productive performance and health of post-weaning piglets.

**Materials and Methods:**

Four regular nutritional phases were prepared. Post-weaning pigs (21–70 days old) received one of two dietary treatments: T1-basal diet (BD-control group) with in-feed antibiotics as a prophylactic medication (one pulse of Tiamulin in P3 and one pulse of Amoxicillin in P4); and T2-BD without in-feed antibiotics but with *Saccharomyces boulardii* RC009 (1 × 10^12^ colony forming unit/T feed). The feed conversion ratio (FCR), total weight gain (TWG–kg), and daily weight gain (DWG–kg) were determined. A post-weaning growth index (GI) was calculated and animals (160 days old) from each treatment were analyzed at the abattoir after sacrifice for carcass weight and respiratory tract lesions.

**Results:**

Pigs consuming probiotics had higher TWG and DWG than the control group. The group of animals with low body weight obtained the same results. *Saccharomyces boulardii* administration decreased diarrhea, and FCR reduction was related to a GI improvement. A significant increase in carcass weight and muscle thickness reduction was observed in animals received the probiotic post-weaning.

**Conclusion:**

*Saccharomyces boulardii* RC009, a probiotic additive, was found to improve the production parameters of pigs post-weaning and enhance their health status, indicating that it may be a promising alternative to prophylactic antibiotics.

## Introduction

During porcine production, the occurrence of infectious diseases negatively impacts production parameters, and the control of disease is thus one of the main strategies for enhancing production [1]. Post-weaning is an especially important and challenging stage in the productive life of a pig, especially if it has a low-weight. For many years, antibiotics have been the main tool used to prevent and contend the damage caused by infectious diseases. Consequently, the routine use of antibiotics in a prophylactic manner is now common in the swine industry [2] and has led to a decrease in therapeutic efficiency and the emergence of microorganisms with reduced antibiotic sensitivity [3]. These antibiotic resistance determinants can be transferred from livestock sources to humans and to the environment through swine farm wastewater. Today, the “one word, one health” concept means that the aim of animal production is to produce safe and high-quality food, while also prioritizing animal and environmental welfare. The intensive use of antibiotics during swine production is thus a critical concern, not only due to the public health risks but also the possible environmental damage [4].

Thus, there is a clear need for innovative strategies for nutritional supplementation to improve the health and production of breeding animals, while also helping to avoid the unnecessary use of antibiotics. Implementing probiotics is one of the most promising and new tools for improving animal health and production [5]. The Food and Agriculture Organization and the World Health Organization have defined them as live microorganisms that, when provided in adequate amounts, can improve the intestinal microbial balance, and confer health benefits to the host [6]. The administration of probiotic additives as natural alternatives to antibiotics to improve intestinal health in productive animals does not produce collateral effects and improves digestibility, average daily gains, and the conversion ratio [7].

Weaning is stressful due to factors such as the separation from the mother and the changes in feed and environment. In extreme cases, this can result in lower feed consumption, diarrhea, decreased conversion efficiency, weight loss, and death [8]. These stress factors are also related to the occurrence of several intestinal and/or systemic pathologies that impact farm performance indicators; their influence on the intestinal gut health of post-weaning pigs impacts lifetime gut health, and consequently, on animal performance [9]. Several studies have evaluated the effects of probiotic use on the productivity of pigs at this stage [7, 10, 11]. Jensen *et al*. [12] evaluated the effects of *Saccharomyces cerevisiae* on preventing diarrhea in weaned piglets by supplementing the diet from prepartum to weaning and in the animals during rearing, and the results were compared with a control group. The data obtained reflected a lower incidence of diarrhea in the treated animals and a lower risk of suffering *Escherichia coli* infections. Studies have shown that probiotic supplementation in swine diets can improve growth, conversion efficiency, nutrient utilization, intestinal health, immune system regulation, carcass weight, and lumbar fat thickness [11, 13]. The probiotic properties of *S. cerevisiae* var. *boulardii* RC009 isolated from the animal ecosystem have demonstrated its ability to survive under gastrointestinal conditions, to aggregate and inhibit pathogenic bacteria, and its capacity to bind mycotoxins [14, 15].

To date, however, little is known about the effects of *Saccharomyces boulardii* with *in vivo* trials, under field conditions, when compared to the routine use of antibiotics, and especially the effects on low-weight piglets. This study aimed to evaluate *S. cerevisiae* var. *boulardii* RC009, as an in feed nutritional additive that can substitute the prophylactic use of antibiotics to improve productive performance and health in post-weaning piglets, with a specific focus on low-weight piglets.

## Materials and Methods

### Ethical approval

The working protocol and the techniques used comply with the regulations of the Subcommittee on Animal Bioethics under the Ethics Committee of Scientific Research, as established in Resolution 376/22 of the Superior Council of the National University of Rio Cuarto.

### Study period and location

The study was conducted from October to December 2022. This experiment was carried out at the Aceitera General Deheza SA pigs farm in Santa Eufemia, Córdoba, Argentina. The rainy season lasts for approximately 10 months, with a sliding 31-day rainfall of at least 13 mm and an average total accumulation of 131 mm. The period of the year without rain lasts approximately 2 months, with an average total accumulation of 8 mm. The temperate season has an average daily maximum temperature of 27°C and an average minimum temperature of 19°C. The cool season has an average daily maximum temperature of <18°C and an average maximum temperature of 15°C.

### Probiotic microorganism identification and biomass production

*Saccharomyces cerevisiae* var. *boulardii* RC009 was isolated from a healthy pig intestine. Morphological, biochemical, and molecular characterizations were conducted according to Armando *et al*. [14]. Species assignment was determined using the Yeast Identification Database (www.yeast-id.com). The sequence comparisons were performed using the basic local alignment search tool program within the National Center for Biotechnology Information database and submitted to GenBank (ID #KF447149.1).

The *S. cerevisiae* var. *boulardii* RC009 biomass was obtained from 24 h cultures in Yeast-Peptone-Dextrose broth, which contained 1 g PO_4_H_2_K/L in a BioFlo 2000 fermenter (New Brunswick Scientific Co., Inc., Enfield, CT, USA) operated at 4× *g* at 28°C, for 12 h, and 1.5 vvm aeration. The pH value was adjusted to 5 with 6 M NaOH and the working volume was 4 L.

### Probiotic additive formulations and diets

The obtained biomass was collected at the end of the fermentation process and centrifuged at 1000× *g* at 4°C for 10 min. The concentrated pellet was resuspended (1:1) with cryoprotectant (10% skim milk plus 5% yeast extract, for the yeast and 10% skim milk only for lactic acid bacteria) and stored at −80°C. The viability of the lyophilized formula (1 g) was confirmed.

Four regular phase nutrition schemes of the farm during post-weaning and independent of the treatment were prepared. The phases 1, 2, 3, and 4 diets (P1, P2, P3, and P4, respectively) were administered for 5, 9, 15, and 7 d, respectively. The P1 and P2 diets were commercially obtained, while P3 and P4 diets were prepared at the farm nutrition plant. They were formulated to meet the requirements of post-weaning suggested by the National Research Council [16]. The nutrient content of the diet was estimated using chemical analysis according to the Association of Official Analytical Chemists [17]. Diet composition is shown in Table-1.

**Table-1:** Centesimal composition and calculated values of diets provided to the animals in the experimental period.

Item	Unit	Phase 1	Phase 2	Phase 3	Phase 4
Dry matter	%	92.01	90.61	88.84	88.76
Crude protein	%	22.70	22.23	19.87	19.99
Metabolizable energy	Kcal	3,676.61	3,595.31	3,429.34	3,402.43
Total lysine	%	1.66	1.60	1.42	0.00
Digestible lysine	%	1.55	1.50	1.31	1.19
Digestible methioine	%	0.61	0.61	0.55	0.45
Digestible cysteine	%	0.33	0.29	0.25	0.26
Digestible Met+Cyst	%	0.93	0.90	0.88	0.00
Digestible tryptophan	%	0.36	0.34	0.24	0.23
Digestible threoine	%	1.01	0.97	0.84	0.77
Digestible arginine	%	1.14	1.19	0.00	0.00
Digestible valine	%	0.64	0.74	0.00	0.00
Crude fat	%	9.15	8.39	4.96	4.71
Crude fiber	%	1.91	2.39	3.49	4.00
Calcium	%	0.86	0.85	0.67	0.81
Total phosphorus	%	0.59	0.57	0.65	0.72
Available phosphorus	%	0.57	0.50	0.44	0.43
Lactose	%	15.00	7.50	0.00	0.00
Linoleic acid (C18:2)	%	2.71	3.07	2.45	0.00
Choline	mg/kg	735.00	735.00	593.22	315.00
Zinc	Ppm	3,000.00	3,000.00	1,639.12	139.12
Copper	Ppm	266.20	266.20	263.50	263.50
Selenium	Ppm	0.40	0.40	0.34	0.34
Iron	Ppm	90.39	90.39	75.47	75.45
Sodium	%	0.46	0.34	0.22	0.17
Chlorine	%	0.51	0.37	0.30	0.24
Ash	%	5.98	5.73	4.62	5.13

### Experimental design

The *in vivo* study was conducted using post-weaning pigs, from 21 to 70 days old. Two dietary treatments were included: T1- experimental diets related to each growth stage phase named basal diet (BD-control group) with in-feed antibiotics as prophylactic medications (one pulse of Tiamulin [7 mg/kg] was provided during the last 10 d of P3 and another pulse of Amoxicillin [20 mg/kg] was administered in P4); and T2-BD without in-feed antibiotics but with *S. boulardii* RC009 (1 × 10^12^ colony forming unit/T feed) supplemented during all experiments. Diets were prepared by premixing the probiotic additive separately with the different diet phases and they were then mixed in an industrial mixer.

The experience was carried out in a multi-site, farrow-to-finish pig farm, with a breeding herd of 2400 sows (Agroceres PIC, Argentina). A total of 1099 piglets (581 male and 518 female), weaned at 21 days of age, were selected from the full cycle experimental farm, with a weaning weight of 6.63 kg ± 0.47 (p ≤ 0.05). From those 1099 animals, two different barns were filled, one with 552 piglets in the control group (T1) and another with 547 animals in the treated group (T2). In each group, the pigs were blocked by body weight (BW) and sex. During the first 2 weeks of the post-weaning, animals with low BW (LBW) or bad body conditions were segregated to a therapy group inside each barn. The piglets were housed in pens with a fully slatted plastic floor. Each pen was equipped with one double-sided wet/dry feeder with one nipple drinker. All pigs had *ad libitum* access to feed and water during the trial period. The light was on daily in the environmental control unit at 7 am and off at 7 pm. The room temperature curve was set between 27°C and 23°C throughout the experiment. On 21 and 42 days of age, the piglets were vaccinated for Porcine Circovirus 2 and *Mycoplasma hyopneumoniae* with the commercial vaccine, and on 50 days of age for *Actinobacillus pleuropneumoniae*.

Furthermore, for a longitudinal study, 20 animals were randomly selected on 21 days of age and tagged in both treatment and control groups. In addition, 10 pigs (five male and five female) were randomly selected and tagged at 40 days old from a subgroup of animals with a lower than average BW (LBW).

### Productive parameter determination

When the piglets arrived at the post-weaning barn, the group was weighed on the truck (Total initial weight), and an average initial weight was registered. Then, the same procedure was performed when the pigs were transferred to the fattening period at 70 days of age (Total final weight and average final weight). In addition, at the end of the post-weaning phase, all animals from each barn were weighed in groups of 10 to evaluate the dispersion of the average weight between treatments.

The amount of feed offered to the full barn/group was recorded at the end of each dietary phase. The summary of all phases was calculated in each barn to estimate the global feed intake (total feed administered-remaining feed). The feed conversion ratio (FCR) (Global Feed Intake: Global Weight Gained) for each treatment (control and treated) was also determined.

Individual weaning Piglets’s weights were recorded in tagged pigs at the beginning of the experiment (W0) and at the end of every phase diet (W1, W2, W3, and W4) until 60 days old. The total weight gain (TWG–kg) and daily weight gain (DWG–kg) were determined for tagged pigs in each treatment (separating the standard animals from the LBW subgroup animals) and for sex at each growing phase. In addition, a post-weaning growth index (GI) was calculated as the coefficient between each individual’s final weight (W4) and initial weight (W0).

At the end of the fattening period, 160 days old animals from each treatment were analyzed at the abattoir after sacrifice, for carcass weight determination and quantification of respiratory tract lesions.

### Hematological analysis

Blood samples (5 mL) were collected from the 32 days old pigs (6) in each replicate (selected randomly), through the anterior vena cava into vacuum blood collection tubes with ethylenediaminetetraacetic acid.

Samples were immediately transported to the laboratory and analyzed to determine their hematological profiles. The erythrocyte (Er) histogram and leukogram were obtained using an automated analysis process (Diatron MI PLC, Budapest, Hungary). The hematological parameters such as (Er millions/μL), hemoglobin (HGB g/dL), hematocrit (Ht%), mean corpuscular volume (fL), mean corpuscular hemoglobin (MCH pg), MCH concentration, total proteins (TP gr/dL), fibrinogen (mg/dL), leukocytes (miles/μL), segmented neutrophils (miles/μL), segmented neutrophil percentage monocyte (Mono) concentration, lymphocytes (Lymph miles/μL), Lymph percentage, (Mono miles/μL), Mono percentage, eosinophils (EOS miles/μL), EOS percentage, and platelets (Plt miles/μL) were determined and quantified.

### Carcass quality determination

After the fattening period, the carcass weight (kg) with head and shanks was recorded at the slaughterhouse. The pig carcass is the entire body of the slaughtered animal as it appears after bleeding and gutting operations, whole or split in half, without bristles, hooves, and genitalia. The width of the *Longissimus thoracis* at the thoracolumbar junction was measured using a Vernier caliper, according to another essay [18]. All these parameters were measured individually (per animal) in each experimental group.

### Pig health status and macroscopic lesion scores

Pigs were clinically revised every weighing day to evaluate the occurrence of cough, sneezing, and diarrhea. The presence or absence of diarrhea (liquid feces on the floor and/or dirty anal region) was determined. In addition, the nasal turbinate and lung lesions were scored at slaughter, in accordance with the previous studies [19, 20].

### Statistical analysis

For the tagged pigs, productive parameters (DWG and TWG) and carcass quality were determined using each animal as an experimental unit. The data were analyzed using a general linear and mixed model with R Statistical software (R Core Team, Vienna, Austria 2021). Means and standard deviation (SD) were compared using the Mann–Whitney-Wilcoxon test (p ≤ 0.01).

## Results

### Productive parameter determination

**Figure-1:**
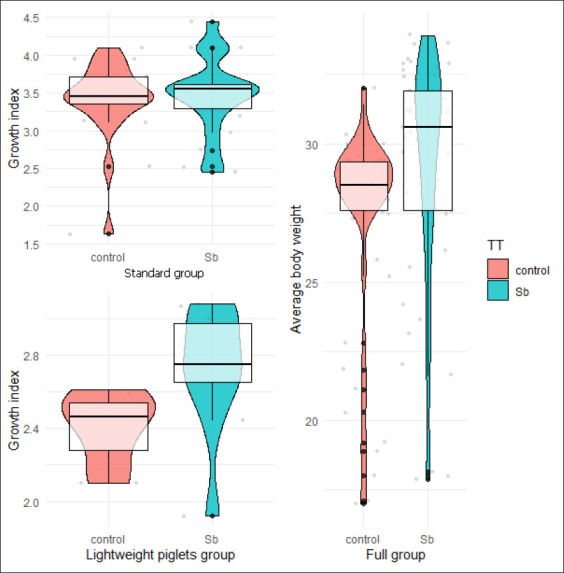
Individual growth index of pigs from 21 to 60 days old in the standard group, and from 40 to 60 days old in the therapy subgroups for both treatments; and average body weight of all animals in the study at 70 days old, weighted in groups of 10.

At the end of the post-weaning phase, when all the barns were weighed (including the LBW group), the average daily gain (ADG) of the treated group was found to be 6 g higher than that of the control group (Table-2). When the pigs were weighed in groups of 10 animals, the average weight was 29.10 (SD = 4.23) in the probiotic-treatment group and 27.29 (SD = 3.7) in the control group (Figure-1), and the differences were statistically significant (p = 0.0007).

**Table-2:** Population productive parameters at the entry and the exit of post-weaning phase.

Variables	Treated with probiotic	Control
Total initial weight	3700	3520
No. of animals	556	558
Average initial weight	6.654	6.308
Total final weight	16100	15880
No. of animals	547	552
Average final weight	29.43	28.7
Global weight gained	12400	12360
Average daily gain	0.463	0.457
Global feed intake	19350	19350
FCR	1.56	1.56

FCR=Feed conversion ratio

For the tagged pigs, the standard and LBW groups that received probiotics did not show any significant differences in relation to sex. The same behavior was observed for the animals that did not receive the probiotics. However, the control therapy females showed higher DWG when compared with the male animals (p ≤ 0.0005, data not shown). Furthermore, the DWG was 440.4 g (SD 97.8) in the control group (T1) and 475.3 g (SD 91.5) in the group consuming the probiotic additive (T2), and no significant differences were observed between groups (p = 0.233). The productive parameters of the weaned piglets from 21 to 60 days old that were fed *S. boulardii* diets at different growth phases are shown in Table-3. The studied parameters for TWG and DWG showed no significant differences between the animals treated with probiotics and in-feed antibiotics (T1-control). A detailed analysis revealed that the probiotic influence on the standard animals mainly occurred during the fourth growth phase until 60 days old (Table-3). The GI showed no significant differences between T1 and T2 in the standard pigs (p = 0.755); however, there were significant differences in the LBW subgroup (p = 0.010) (Figure-1).

**Table-3:** Productive parameters of tagged weaned pigs fed *Saccharomyces boulardii* supplemented diets.

Growth phases	Productive parameters (mean ± SD)				p-value

	Control group		Supplemented group		
	
	W (kg)	DWG (g)	W (kg)	DWG (g)	
0 (W0)	6.24 (0.32)		6.77 (0.38)		
1 (W1)	7.19 (0.55)	157.5 (87.8)	7.56 (0.50)	197.6 (114.5)	0.189
2 (W2)	9.49 (1.31)	328.1 (168.2)	10.85 (1.41)	298.7 (123.8)	0.218
3 (W3)	17.80 (2.85)	593.3 (127.8)	18.10 (2.27)	557.5 (96.7)	0.107
4 (W4)	21.22 (3.36)	489.3 (162.8)	23.41 (3.18)	759.1 (240.4)	<0.001
TWG	14.97 (3.32)		16.64 (3.20)		

TWG=Total weight gain, DWG=Daily weight gain, SD=Standard deviation. Wilcox test p-values for differences in DWG between groups

### Hematological analysis

The effects of the probiotic *S. boulardii* RC009 supplementation on the hematological parameters is shown in Table-4. Higher values were found in some red blood cell parameters in the treated group, such as Ht% and HGB, but also in the TP, Mono, EOS, and Plt. However, there were no significant differences between the groups (p > 0.05).

**Table-4:** Effect of probiotic *Saccharomyces boulardii* RC009 supplementation on hematological parameters.

Hematological parameters	Control (mean ± SD)	*Saccharomyces boulardii* RC009 (mean ± SD)
Er	6392222 ± 504607.17	6589411.8 ± 451982.20
Hgb	10.19 ± 0.67	10.8 ± 0.63
Ht	32.39 ± 2.33	35.28 ± 2.05
MCV	48.83 ± 11.19	55.0 ± 3.82
MCH	15.99 ± 1.18	16.46 ± 1.44
MCHC	30.93 ± 0.77	30.59 ± 0.82
TP	4.67 ± 0.32	5.12 ± 0.29
Fib	272.22 ± 98.91	305.88 ± 151.35
LEK	20350 ± 3007.99	19482.35 ± 5209.52
Neut (%)	39.78 ± 11.38	40.35 ± 8.8
Neut	8214.22 ± 3322.63	7960.47 ± 3417.16
Lynph (%)	55.33 ± 11.85	53.0 ± 11.10
Lynph	11096.72 ± 2447.11	10129.47 ± 2634.99
Mono (%)	2.13 ± 0.96	4.68 ± 2.88
Mono	452.53 ± 229.96	982.0 ± 863.59
Eos (%)	2.07 ± 1.44	2.77 ± 1.72
Eos	424.28 ± 305.05	554.46 ± 333.50
Plt	414000 ± 158676.47	552764.71 ± 266624.99

Er millions/µL=Erythrocytes, Hgb gr/dL=Haemoglobin, Ht %=Haematocrit, MCV fl=Mean corpuscular volume, MCH pg=Mean corpuscular haemoglobin, MCHC %=Mean corpuscular haemoglobin concentration, TP gr/dL=Total proteins, Fib mg/dL=Fibrinogen, LEK miles/µL=Leukocytes, Neut miles/µL=Segmented neutrophils, Neut %=Segmented Neutrophils percentage concentration, Lynph miles/µL=Lymphocytes, Lynph %=Lymphocytes percentage, Mono miles/µL=Monocytes, Mono %=Monocytes percentage, Eos miles/µL=Eosinophils, Eos %=Eosinophils percentage and Plt miles/µL=Platelets. There were no significant differences (p>0.05), SD=Standard deviation

### Carcass weight and muscle thickness

A significant difference was observed in the weight of the carcass and in the thickness of the muscle, after the animals finished their fattening period and went to the slaughterhouse. The carcass weight and muscle thickness results from the probiotic and control treatments determined during the final period and at the slaughterhouse are shown in Table-5.

**Table-5:** Carcass weight and muscle thickness of treatments at the slaughterhouse.

Treatments	Carcass weight (mean ± SD)	Muscle thickness (mean ± SD)
Control	99.8 ± 7.5^a^	81.1 ± 5.1^a^
*Saccharomyces boulardii*	109.0 ± 8.3^b^	86.3 ± 5.0^b^

Different letters indicate significant differences according to the fishers least significant difference test (p ≤ 0.05). Each period must be compared between treatments. SD=Standard deviation

### Pig health status and macroscopic lesion scores

The rate of pneumonia was slightly higher in the control group, but no differences or trends were identified in the presentation of lung or nasal cavity lesions for the pigs in the treated group compared with the control group. The effects of the probiotic *S. boulardii* RC009 supplementation on macroscopic lesion scores at the end of the experimental period are shown in Table-6.

**Table-6:** Effect of probiotic *Saccharomyces boulardii* RC 009 supplementation on clinical signs and macroscopic lesions scores at the slaughterhouse.

Macroscopic lesions scores												

Treatments	Lung pneumonia (%)							P index	Nasal turbinate atrophy index			
	
	LA	RA	LM	RM	I	LC	RC		LD	LV	RD	RV
Control	0.36	1.25	0.76	2.04	0.73	0.59	1.49	1.17	0.80	2.76	0.95	2.66
*Saccharomyces boulardii* RC009	0.53	1.54	0.66	1.63	0.62	0.4	0.50	1.00	1.25	3.0	1.94	2.52

Lung lesions (by lung lobe): LA=Left anterior lobe, RA=Right anterior lobe, LM=Left middle lobe, RM=Right middle lobe, I=Intermediate lobe, LC=Left caudal lobe, RC=Right caudal lobe, Nasal turbinate (by turbinate): LD=Left dorsal turbinate, LV=Left ventral turbinate, RD=Right dorsal turbinate, RV=Right ventral turbinate

## Discussion

The use of the probiotic *S. cerevisiae* var. *boulardii* RC009 as an in-feed nutritional additive to substitute the prophylactic use of antibiotics while maintaining the productive parameters and health of post-weaning pigs were evaluated. The influence of the probiotic on animals with an LBW was also determined.

The pigs that consumed the probiotic additive had a higher TWG, with an average of more than 1 kg greater than that of the control group. Similar weight dispersions were also found between groups. The results are in line with the previous research by Zhang [10], which investigated the rearing of pigs that consumed *S. boulardii*. The authors associated this improved performance with the decreased levels of pro-inflammatory cytokines in the jejunum and ileum, such as TNF-α and IL-6. Furthermore, they also found a higher concentration of serum antioxidants, such as total superoxide dismutase, which could contribute to the protection of the intestinal mucosa. The administration of *S. boulardii* in the diets of the post-weaning pigs also reduced their rates of diarrhea.

A previous study [13] reported that pigs supplemented in the post-weaning phase with an additive including *S. boulardii* reported no difference in DWG but improved FCR in the supplemented group. In the present study, there were no variations in the FC; however, the treated group was found to have a slight increase in DWG but the effects on the productive parameters did not show any statistically significant differences. It is of note though that the minimal increase could represent a significant production increase for the farm. Even the lack of difference between the productive values of the groups could be considered beneficial because of the no use of no-therapeutic antibiotics.

In this investigation, the individual results by feed stages were analyzed and the differences between the treated and control groups were more evident at the final stage of the study, as the probiotic-supplemented animals showed significant differences in DWG when compared with the control group. This variation in the effects according to the age of the animals has previously been reported [13]. Furthermore, pigs consuming the probiotic additive had more dispersion in the ABW than the controls. This was probably due to the need for probiotic habituation or initial microbiota regulation in the first phase of additive consumption.

Weaning lightweight piglets is a common challenge on farms, as these animals have lower productive indexes when compared with heavier animals [21] and require specific facilities and management to achieve acceptable productive indexes. Furthermore, they also represent a hazard to the farms’ sanitary status due to their increased susceptibility to disease. In the present study, pigs were segregated in the first 2 weeks because of low-weight and consumption of the probiotic was found to result in a markedly higher GI when compared with the control group. For the hematological values, the animals that consumed the probiotic additive showed an increase in the values associated with the red line, such as red blood cells and HGB, but also in indicators associated with immunity, such as Mono, EOS or plasma proteins, and even Plt. In another study using a yeast wall feed supplement, animals consuming the additive showed lower levels of neutrophils, Lymph, and Mono than the treated pigs [22]. An increased EOS count was previously reported in pigs that consumed prebiotics through their feed [23]. There may be many reasons for the increase in EOS, including the presence of allergens. Although the increase does not seem to be significant, or recurrent, it may help to generate new studies on the possible effects of these additives on the surrounding EOS populations.

The gut-lung axis has been proposed as a specific axis with interactions between the two microbiotas involved in host health and disease [24].

The absence of differences in respiratory lesions could be related to the study design, as there was a long period between additive consumption and evaluation of the lesions at the slaughterhouse. While the significant increase in carcass weight and reduction in muscle thickness in animals receiving the probiotic during their post-weaning period supports the idea [9] that interventions made during the early stages of animal growth could impact the entire production cycle. Future studies are needed to evaluate the use of biological additives during the fattening period.

## Conclusion

*Saccharomyces boulardii* RC009 is a probiotic additive that can improve the production parameters of pigs during their post-weaning phase and enhance their health status. This probiotic additive is thus a promising alternative to prophylactic antibiotic use.

## Authors’ Contributions

JP, AM, AC, and LC: Designed the study. JP, AM, MCI, VP, AF, and MPM: Carried out the laboratory and fieldwork. JP, AM, and LC: Statistical analysis and drafted the manuscript. AC, MCI, and MPM: Helped in manuscript preparation. All authors have read, reviewed, and approved the final manuscript.
